# A phase I dose escalation study of oxaliplatin plus oral S-1 and pelvic radiation in patients with locally advanced rectal cancer (SHOGUN trial)

**DOI:** 10.1186/s13014-015-0333-8

**Published:** 2015-01-23

**Authors:** Soichiro Ishihara, Satoshi Matsusaka, Keisaku Kondo, Hisanaga Horie, Keisuke Uehara, Masahiko Oguchi, Keiko Murofushi, Masashi Ueno, Nobuyuki Mizunuma, Taijyu Shinbo, Daiki Kato, Junji Okuda, Yojiro Hashiguchi, Masanori Nakazawa, Eiji Sunami, Kazushige Kawai, Hideomi Yamashita, Tohru Okada, Yuichi Ishikawa, Toshifusa Nakajima, Toshiaki Watanabe

**Affiliations:** Department of Surgical Oncology, University of Tokyo, 7-3-1, Hongo, Bunkyo-ku, Tokyo 113-8655 Japan; Department of Gastroenterology, Cancer Institute Hospital, 3-8-31, Ariake, Koto-ku, Tokyo 135-8550 Japan; Department of General & Gastroenterological Surgery, Osaka Medical College, 2-7, Daigaku-cho, Takatsuki, Osaka 569-8686 Japan; Department of Surgery, School of Medicine Jichi Medical University, 3311-1, Yakushiji, Shimotsuke, Tochigi 329-0498 Japan; Division of Surgical Oncology, Department of Surgery, Nagoya University, 65, Tsurumai-cho, Syowa-ku, Nagoya, Aichi 466-8550 Japan; Department of Radiation Oncology, Cancer Institute Hospital, 3-8-31, Ariake, Koto-ku, Tokyo 135-8550 Japan; Department of Gastroenterological Surgery, Cancer Institute Hospital, 3-8-31, Ariake, Koto-ku, Tokyo 135-8550 Japan; Department of Radiology, Osaka Medical College, 2-7, Daigaku-cho, Takatsuki, Osaka 569-8686 Japan; Department of Radiology, Teikyo University, 2-11-1, Kaga, Itabashi-ku, Tokyo 173-8605 Japan; Department of Surgery, Teikyo University, 2-11-1, Kaga, Itabashi-ku, Tokyo 173-8605 Japan; Department of Radiology, School of Medicine Jichi Medical University, 3311-1, Yakushiji, Shimotsuke, Tochigi 329-0498 Japan; Department of Radiology, University of Tokyo, 7-3-1, Hongo, Bunkyo-ku, Tokyo 113-8655 Japan; Department of Radiology, Nagoya University, 65, Tsurumai-cho, Syowa-ku, Nagoya, Aichi 466-8560 Japan; Japanese Foundation for Cancer Research, 3-8-31, Ariake, Koto-ku, Tokyo 135-8550 Japan; Japan Clinical Cancer Research Organization, 7F Ginza Wing Bldg. 1-14-5, Ginza, Chuo-ku, Tokyo 104-0061 Japan

**Keywords:** Rectal cancer, Chemoradiotherapy, Phase I study, Oxaliplatin, S-1

## Abstract

**Background:**

The objective of this phase I study was to determine the maximum tolerated dose (MTD) and recommended dose (RD) of preoperative chemoradiotherapy (CRT) with S-1 plus oxaliplatin in patients with locally advanced rectal cancer.

**Methods:**

Patients received radiotherapy in a total dose of 50.4 Gy in 28 fractions. Concurrent chemotherapy consisted of a fixed oral dose of S-1 (80 mg/m^2^/day) on days 1–5, 8–12, 22–27, and 29–33, plus escalated doses of oxaliplatin as an intravenous infusion on days 1, 8, 22, and 29. Oxaliplatin was initially given in a dose of 40 mg/m^2^/week to three patients. The dose was then increased in a stepwise fashion to 50 mg/m^2^/week and the highest dose level of 60 mg/m^2^/week until the MTD was attained.

**Results:**

Thirteen patients were enrolled, and 12 received CRT. Dose-limiting toxicity (DLT) occurred in two of six patients (persistent grade 2 neutropenia, delaying oxaliplatin treatment by more than 3 days) at dose level 3; there were no grade 3 or 4 adverse events defined as DLT. The RD was 60 mg/m^2^/week of oxaliplatin on days 1, 8, 22, and 29. Twelve patients underwent histologically confirmed R0 resections, and two out of six patients (33%) given dose level 3 had pathological complete responses.

**Conclusions:**

The RD for further studies is 80 mg/m^2^ of S-1 5 days per week plus 60 mg/m^2^ of oxaliplatin on days 1, 8, 22, and 29 and concurrent radiotherapy. Although our results are preliminary, this new regimen for neoadjuvant chemoradiotherapy is considered safe and active.

**Trial registration:**

This trial was registered with Clinicaltrials.gov (identifier: NCT01227239).

## Background

Recent advances in surgical techniques, including procedures such as total mesorectal excision (TME), and recognition of the importance of obtaining an adequate circumferential resection margin (CRM) have improved outcomes in patients with rectal cancer [1,2]. However, even after optimal surgery, the 5-year survival rate of patients with advanced rectal cancer remains between 40% and 60%. Preoperative chemoradiotherapy (CRT) has become a standard treatment option for locally advanced rectal cancer. Achievement of a pathological complete response (pCR) and a negative CRM correlate with good outcomes and are considered early markers of the response to CRT [3-5].

CRT with continuous infusion of 5-fluorouracil (5-FU) has been recommended in addition to surgery to lower the risk of local recurrence as compared with surgical resection alone in patients with locally advanced rectal cancer. Treatment with 5-FU sometimes requires hospitalization or central venous access, because continuous infusion is more effective and less toxic than bolus injections. Oral fluoropyrimidine preparations, such as uracil and tegafur (UFT) or capecitabine, have been substituted for 5-FU in several clinical trials of preoperative CRT and have demonstrated equivalent efficacy and less toxicity than intravenous 5-FU. S-1 is an oral anticancer drug that combines tegafur (a prodrug of 5-FU) with 5-chloro-2, 4-dihydropyridine (CDHP: gimeracil) and potassium oxonate (Oxo). S-1 is unique because it increase the blood concentration of 5-FU and reduces gastrointestinal toxicity due to the action of CDHP and Oxo, respectively. S-1 has shown high antitumor activity and good compliance in patients with gastrointestinal cancer and other solid tumors [6]. In addition, CDHP has also been shown to enhance the antitumor activity of irradiation in human colon cancer xenograft models [7]. Sadahiro et al. found that concurrent CRT with S-1 was an effective and well-tolerated regimen for locally advanced rectal cancer [8].

Oxaliplatin is a relatively new cytotoxic agent, and combination chemotherapy with a fluoropyrimidine and oxaliplatin has become a standard regimen for metastatic colorectal cancer [9,10]. In an adjuvant setting, the addition of oxaliplatin to 5-FU/LV significantly improves disease-free survival [11,12]. Oxaliplatin has also been suggested to act as a radiosensitizer [13]. A combination of oxaliplatin plus S-1 has been shown to be a feasible regimen with promising antitumor activity in patients with metastatic colorectal cancer [14].

These results of previous studies have thus suggested that clinical trials of chemotherapy with S-1 and oxaliplatin plus radiotherapy are warranted in patients with locally advanced rectal cancer. In this phase I, dose-finding study, patients with rectal cancer received escalating doses of oxaliplatin combined with fixed doses of S-1 and pelvic radiotherapy. The primary objective was to determine the maximum tolerated dose (MTD) and recommended dose (RD) of oxaliplatin given as an intravenous infusion in combination with oral S-1 and preoperative pelvic radiotherapy. Secondary objectives included the evaluation of safety, compliance, and preliminary efficacy.

## Methods

### Eligibility criteria

Patients between 20 and 75 years of age who had histologically confirmed, non-metastatic, primary adenocarcinoma (well or moderately differentiated adenocarcinoma) of the middle or lower rectum (cT3-T4, any N, M0) were eligible for enrollment. Disease was staged according to the staging system of the American Joint Committee on Cancer, sixth edition. Additional eligibility criteria included a T stage of T3 or T4 on computed tomography (CT) plus magnetic resonance imaging (MRI); a resectable tumor as prospectively defined by the surgeon in charge; good general condition enabling major surgery (Eastern Cooperative Oncology Group performance status 0 or 1); normal liver, renal, and bone marrow functions; and written informed consent from the patient. Exclusion criteria were as follows: prior chemotherapy for rectal cancer or any prior pelvic irradiation; a history of malignant disease; severe heart disease, uncontrolled infection, or metabolic disorders; or severe neurologic impairment or inflammatory bowel disease. This trial was approved by institutional review boards of all participating centers. All patients provided written informed consent before undergoing study-specific screening procedures. The trial was registered with Clinicaltrials.gov (identifier: NCT01227239).

### Radiotherapy

The radiation therapy protocol guidelines in concurrent chemotherapy were defined as followings. 1) The 50.4 Gy of preoperative radiation therapy was delivered in 28 fractions over approximately 6 weeks with 10-MV photons or greater. 2) The Three dimensional conformal radiotherapy (3D-CRT), such as 3 fields technique or 4 fields box technique, was required, however, the intensity modulated radiotherapy (IMRT) was not allowed. 3) The following clinical target volume (CTV) were encompassed with treatment volume (PTV) of 3D-CRT, namely, the gross tumor volume (GTV) with appropriate margin, mesorectum, presacral lymph nodes, bilateral obturator lymph nodes, and bilateral internal iliac lymph nodes up to the level of promontory.

### Chemotherapy

S-1 was given orally twice daily (after breakfast and dinner) on days 1–5, 8–12, 22–27, and 29–33. The dose of S-1 was fixed at 80 mg/m^2^/day. Oxaliplatin was given as an intravenous infusion in three escalating dose levels. The initial dose of oxaliplatin was 40 mg/m^2^ (level 1), 33% reduction of the approved dose, the intermediate dose 50 mg/m^2^ (level 2), and the maximum dose 60 mg/m^2^ (level 3), the upper limit of the approved dose, given on days 1, 8, 22, and 29. If DLT occurred at level 1, the dose of S-1 was reduced to 65 mg/m^2^/day (level 0), and the dose of oxaliplatin was reduced to 40 mg/m^2^.

### DLT

Adverse events were classified according to the Common Terminology Criteria for Adverse Events (CTCAE), version 4. DLT was defined as grade 4 neutropenia or anemia; grade ≥3 febrile neutropenia or thrombocytopenia; grade ≥3 nonhematologic toxicity (excluding anorexia, nausea, and vomiting); a delay of more than 3 days in oxaliplatin administration; withholding 13 or more of the 40 scheduled doses of S-1 because of toxicity; or the interruption of radiation for 1 week or longer because of toxicity.

### MTD and RD

Three patients were assigned to the first cohort (level 1), and their eligibility was checked by the patient registration center. Eligible patients started to receive chemotherapy and radiotherapy as described above, after reconfirming that they met all criteria for starting the protocol treatment. The patients were observed for adverse events up to 28 days after termination of CRT. If none of the first three patients had DLT during the treatment or observation periods, three additional patients were recruited and assigned to the next cohort, which received level 2. If all three patients had DLT, level 1 was defined as MTD. If one or two of the first three patients had DLT, the cohort was expanded to six patients at the same dose level. If only one or two of the six patients had DLT, a new cohort of patients proceeded to the next dose level. If three or more of four, five, or six patients had DLT, this dose level was defined as the MTD. The RD for subsequent phase II studies was defined as the level one step below the MTD.

### Evaluation of response

Surgery was performed between 6 and 10 weeks after the completion of CRT. The surgical procedure included a low anterior resection and an abdominoperineal resection, performed by mesorectal resection or tumor-specific mesorectal excision techniques.

The results of operation were classified as R0 if the resection margin was free of tumor, R1 if the tumor was macroscopically removed, but tumor was present at the resection margin, or R2 if tumor clearly remained in the operative field on macroscopic examination. A pathological tumor response was defined as a pCR. An experienced pathologist evaluated specimens for pCR according to the pathologic Tumor Regression Grade (TRG). The TRG was classified according to the Japanese Guidelines for Clinical and Pathological Studies on Carcinoma of the Colorectum, 7th edition. Downstaging was defined as any reduction in the pathological T or N stage after operation as compared with the clinical T or N stage before starting treatment. Patients were observed for complications up to 28 days after operation.

## Results

Between September 2010 and September 2011, a total of 13 patients were enrolled in this phase I study at five hospitals in Japan. Twelve patients received the protocol treatment; one patient whose clinical laboratory data did not meet the criteria for starting CRT was withdrawn. The patient and tumor characteristics at baseline are summarized in Table 1.

**Table 1:** **Patient and tumor characteristics at baseline (n = 12)**

**Characteristic**	**No. of patients**
Median age, year (range)	62, (31–74)
Gender: Male/Female	10/2
ECOG PS: 0/1	12/0
Clinical T classification	
cT3/cT4	12/0
Clinical N classification	
cN0/cN+	3/9
Clinical stage	
cStage IIA/IIIA/IIIB/IIIC	3/0/8/1
Histologic differentiation	
Well/Mod*	7/4

*Well: well differentiated adenocarcinoma, Mod: moderately ;differentiated adenocarcinoma.

### DLT and RD

There was no DLT in patients who received dose levels 1 or 2. At dose level 3 (oxaliplatin 60 mg/m^2^), two of the first three patients had DLT: in both patients oxaliplatin administration had to be delayed by more than 3 days because of grade 2 neutropenia. Therefore, three additional patients received this dose level. None of these patients had DLT. Although level 3 did not reach the MTD, dose escalation was stopped at 60 mg/m^2^ of oxaliplatin because this was the highest level planned. Consequently, 60 mg/m^2^ of oxaliplatin (dose level 3) was designated as the RD for phase II studies in combination with 80 mg/m^2^ of S-1 twice daily and concurrent radiotherapy.

### Other adverse events

Table 2 lists the most common clinical adverse events and laboratory abnormalities according to dose level. Overall, leukopenia was the most frequent adverse event, occurring in 8 patients (67%); however, it was manageable in all patients. Grade 3 or 4 adverse events were infrequent, and only one patient (8%) had grade 3 neutropenia at level 3. No patient had grade 3 or 4 nonhematologic toxicity.

**Table 2:** **Toxicity profiles according to oxaliplatin dose levels**

	**Level 1 (n = 3)**		**Level 2 (n = 3)**		**Level 3 (n = 6)**	
**NCI-CTCAE, version 3**	**G1/2**	**G3/4**	**G1/2**	**G3/4**	**G1/2**	**G3/4**
Leukopenia	0/2	0/0	1/0	0/0	1/3	1/0
Neutropenia	0/1	0/0	0/0	0/0	0/2	1/0
Anemia	1/0	0/0	2/0	0/0	4/0	0/0
Thrombocytopenia	3/0	0/0	1/0	0/0	2/1	0/0
AST abnormalities	3/0	0/0	1/0	0/0	2/0	0/0
ALT abnormalities	2/1	0/0	1/0	0/0	3/0	0/0
Hyperbilirubinemia	0/0	0/0	0/1	0/0	1/0	0/0
Anorexia	0/0	0/0	1/2	0/0	1/0	0/0
Nausea	0/0	0/0	0/1	0/0	1/0	0/0
Fatigue	0/0	0/0	1/0	0/0	1/0	0/0
Alopecia	0/0	0/0	1/0	0/0	0/0	0/0
Diarrhea	2/0	0/0	1/1	0/0	3/0	0/0
Anorectal pain	0/0	0/0	0/0	0/0	0/0	0/0
Abdominal pain	0/0	0/0	1/0	0/0	0/0	0/0
Vein inflammation	0/0	0/0	0/2	0/0	0/0	0/0
Weight loss	1/1	0/0	1/0	0/0	0/0	0/0
Radiation rash	2/0	0/0	0/1	0/0	3/2	0/0
Sensory neuropathy	0/0	0/0	0/0	0/0	1/0	0/0
Hand-foot syndrome	0/0	0/0	0/0	0/0	0/0	0/0

### Compliance

There was full compliance (no reduction in the planned dose or duration of treatment) with radiation therapy in the patients who received dose levels 1 and 2. Of the six patients given dose level 3, two required interruptions of CRT. All patients underwent anterior resection with TME. There was no treatment-related death. Only one patient given dose level 3 had anastomotic leakage requiring subcutaneous drainage. No other serious postoperative complications occurred.

### Tumor response

Twelve (100%) patients underwent R0 resection. No patient at dose level 1 or 2 had a pCR; however, a pCR was achieved in two patients (33.3%) at dose level 3. At dose level 1, two patients (66.7%) had T downstaging, and none had N downstaging. The combined pathological downstaging rate was 66.7%. At dose level 2, three patients (100%) had T downstaging, and one (33.3%) had N downstaging for a combined pathological downstaging rate of 100%. At dose level 3, three patients (50%) had T downstaging, and 5 (83.3%) had N downstaging for a combined pathological downstaging rate of 83.3% (Table 3).

**Table 3:** **Tumor response**

	**Level 1 (n = 3)**	**Level 2 (n = 3)**	**Level 3 (n = 6)**	**Total (n = 12)**
pCR rate	0% (0/3)	0% (0/3)	33.3% (2/6)	16.7% (2/12)
				(95% CI: 4.70-44.80)
R0 rate	100% (3/3)	100% (3/3)	100% (6/6)	100% (12/12)
				(95% CI: 75.8-100)
T downstaging (cT > pT)	66.7% (2/3)	100% (3/3)	50% (3/6)	66.7% (8/12)
N downstaging (cN > pN)	0.0% (0/3)	33.3% (1/3)	83.3% (5/6)	50% (6/12)
Down-staging	66.7% (2/3)	100% (3/3)	83.3% (5/6)	83.3% (10/12)
				(95% CI: 55.2-95.3)

## Discussion

A review of preoperative CRT reported that pCR correlates with good outcomes [15]. Hartley et al. demonstrated that the incidence of pCR after CRT for rectal cancer depends on the mode of delivery of 5-FU, the use of a two-drug regimen, and the dose of radiotherapy [16]. Preoperative CRT including new agents is thus being actively investigated to improve patient outcomes. Intensified neoadjuvant CRT with a fluoropyrimidine and oxaliplatin is being studied in rectal cancer. 5-FU is the most widely used radiosensitizer, but protracted, continuous 5-FU infusion has potential problems associated with the use of central venous catheters, such as infection and deep vein thrombosis. From this point of view, oral drug therapy is safer and more convenient than infusional 5-FU. The oral fluoropyrimidine capecitabine has been studied in combination with radiotherapy in rectal cancer, using different doses and application times. Continuous (7 days/week) or intermittent treatment with capecitabine (5 days/week followed by 2 days’ rest or 14 days followed by 7 days’ rest) has been recommended in combination with preoperative radiotherapy (RT). The total dose of capecitabine delivered by this regimen is higher than 80,000 mg/m^2^, which is equivalent to 70% to 90% of the dose recommended for capecitabine monotherapy. Phase I studies of capecitabine plus radiotherapy have established dose levels in the range of 800 to 900 mg/m^2^, given twice daily throughout radiotherapy. The optimal schedule for S-1 combined with radiotherapy had not been fully evaluated in rectal cancer. S-1 is generally administered according to an intermittent schedule of 80 mg/m^2^/day for 4 weeks, followed by 2 weeks of rest. The intermittent treatment schedule used in our study to administer S1 (5 days’ treatment followed by 2 days’ rest) has the potential to increase dose intensity, enhance tolerability, and improve efficacy. The total administered dose of S-1 has nearly the same potential as the recommended dose of capecitabine [17].

Wong et al. performed a randomized phase II study of neoadjuvant capecitabine and irinotecan or capecitabine and oxaliplatin with concurrent radiotherapy in patients with locally advanced rectal cancer [18]. Preoperative CRT with capecitabine plus oxaliplatin has clinically significant activity (10 pCRs among 48 patients) and acceptable toxicity. In contrast, frequent treatment with irinotecan plus S-1 carries an increased risk of diarrhea [19]. Choi et al. reported that 13% of patients who received irinotecan and S-1 combined with radiotherapy had grade 3 diarrhea [20]. These results suggest that oxaliplatin plus S-1 is better tolerated than irinotecan plus S-1.

In previous trials which have evaluated oxaliplatin in combination with fluoropyrimidine-based CRT [21-24], fluoropyrimidine plus oxaliplatin was not significantly superior to fluoropyrimidine alone. In these studies, the rates of grade 3–4 toxicity were significantly higher in the fluoropyrimidine plus oxaliplatin group than in the fluoropyrimidine alone group, and compliance with all components of preoperative CRT was remarkably lower in the former. The lack of a beneficial effect of adding oxaliplatin to fluoropyrimidine-based CRT was mainly attributed to increased acute toxic effects and complications, resulting in poor compliance with all components of preoperative CRT, including radiotherapy. In these negative studies, a washout period, or a “chemotherapy gap”, was not incorporated in the regimen of chemotherapy given with radiotherapy. A “chemotherapy gap” might reduce toxicity, and promote compliance, and increase efficacy of CRT. The CRT regimen used in this SHOGUN trial included a “chemotherapy gap” in the third week of radiotherapy. In fact, the CAO/ARO/AIO-04 phase III trial [24,25], which also included a “chemotherapy gap”, showed that the addition of oxaliplatin to infusional 5-FU contributed to improved pCR and disease-free survival at 3 years as compared with 5-FU alone, with no significant difference in grade 3–4 toxicities or postoperative complications. The better response in the fluoropyrimidine plus oxaliplatin group was ascribed to the excellent compliance rates. Rodel et al. claimed that the incorporation of a “chemotherapy gap” in the third week of radiotherapy apparently promoted compliance with all components of preoperative CRT. Therefore, the benefit of including a chemotherapy gap should be further studied.

In Phase I or II trials of preoperative CRT with capecitabine and oxaliplatin in patients with locally advanced rectal cancer, pCR rates appeared to be at least twice as high as those achieved with conventional 5-FU-based regimens (10% to 24%) [26-28] (ref.). In a Korean phase I study, the doses of oxaliplatin (50 mg/m^2^) and radiotherapy were fixed, and the dose of S-1 was escalated. The initial dose of S-1 was 50 mg/m^2^/day (level 1). The dose was then escalated to 60 mg/m^2^/day (level 2), 70 mg/m^2^/day (level 3), and 80 mg/m^2^/day (level 4) [29]. The pCR rate was 22.9% in a Korean phase II study of S-1 80 mg/m^2^ and oxaliplatin 50 mg/m^2^ [29]. This pCR rate did not differ significantly from the rates obtained with capecitabine with oxaliplatin. The dose of oxaliplatin might not have reached the most effective dose in the Korean Phase II trial. In our study, the doses of S-1 (80 mg/m^2^) and radiotherapy were fixed, and dose of oxaliplatin was escalated according to a predetermined schedule. No patient (0%) had a pCR at dose level 1 (40 mg/m^2^/day oxaliplatin) or 2 (50 mg/m^2^/day oxaliplatin), but 2 patients (33%) who received dose level 3 (60 mg/m^2^/day) had pCRs. There was a significant difference in the rate of pCR between level 1 or 2 and level 3. In terms of efficacy, 60 mg/m^2^/week oxaliplatin was most effective.

The safety profile of oxaliplatin plus oral S-1 and radiotherapy with a “chemotherapy gap” was found to be good, and treatment-related toxic effects were manageable in all patients. The relatively high rate of neutropenia may be related to high 5-FU concentrations in plasma. Grade 3 or 4 diarrhea did not occur in this study. The potassium oxonate component of S-1 may have contributed to the low rate of gastrointestinal toxicity by inhibiting phosphorylation of 5-FU in the gut. Hand-foot syndrome, commonly associated with 5-FU infusion and capecitabine, did not occur in our study. Sensory neuropathy, a common adverse effect of oxaliplatin, also did not develop. Surgical complications after intensified CRT continue to be a major concern. One patient in our series had anastomotic leakage requiring subcutaneous drainage. In the German rectal cancer trial, the rate of anastomotic leakage of any grade was 11% in the preoperative treatment group. Because our study group was small, patients should be closely monitored for surgical complications in further phase II clinical trials. With regard to compliance, the results of our study were very satisfactory.

## Conclusions

Our results suggest that neoadjuvant CRT combining 60 mg/m^2^/week of oxaliplatin (on days 1, 8, 22, and 28) and 80 mg/m^2^/day of S-1 (on days 1–5, 8–12, 22–27, and 29–33) with 50.4 Gy preoperative radiotherapy is a promising regimen for locally advanced rectal cancer. Our findings indicate that S-1 may become a valuable option, along with capecitabine or 5-FU, for with the management of rectal cancer. On the basis of our results, a multicenter phase II study of preoperative CRT with S-1 plus oxaliplatin is now ongoing to investigate the efficacy of this regimen, including outcomes such as pCR rate and long-term survival.

## Abbreviations

TME: Total mesorectal excision
CRM: Circumferential resection margin
CRT: Chemoradiotherapy
pCR: pathological complete response
5-FU: 5-fluorouracil
UFT: Uracil and tegafur
DLT: Dose-limiting toxicity
LV: Leucovorin
MTD: Maximum tolerated dose
RD: Recommended dose
CT: Computed tomography
MRI: Magnetic resonance imaging
3D-CRT: Three dimensional conformal radiotherapy
IMRT: Intensity modulated radiotherapy
CTV: Clinical target volume
GTV: Gross tumor volume
CTCAE: Common Terminology Criteria for Adverse Events
TRG: Tumor Regression Grade
